# Influence of Xenogeneic and Alloplastic Carriers for Bone Augmentation on Human Unrestricted Somatic Stem Cells

**DOI:** 10.3390/ma15144779

**Published:** 2022-07-07

**Authors:** Lara Schorn, Anna Sine, Karin Berr, Jörg Handschel, Rita Depprich, Norbert R. Kübler, Christoph Sproll, Majeed Rana, Julian Lommen

**Affiliations:** 1 Department of Oral-, Maxillofacial and Facial Plastic Surgery, University Hospital Düsseldorf, Moorenstr. 5, 40225 Düsseldorf, Germany; anna.sine@yahoo.de (A.S.); karin.berr@med.uni-duesseldorf.de (K.B.); rita.depprich@med.uni-duesseldorf.de (R.D.); kuebler@med.uni-duesseldorf.de (N.R.K.); christoph.sproll@med.uni-duesseldorf.de (C.S.); rana@med.uni-duesseldorf.de (M.R.); julian.lommen@med.uni-duesseldorf.de (J.L.); 2 Medical School, Heinrich-Heine-University, Universitätsstr. 1, 40225 Düsseldorf, Germany; info@klinikamkaiserteich.de; 3 Klinik am Kaiserteich, Reichsstraße 59, 40217 Düsseldorf, Germany

**Keywords:** bone augmentation, dental bone substitute materials, xenogeneic bone grafts, alloplastic bone grafts, unrestricted somatic stem cells, bone regeneration

## Abstract

Alloplastic and xenogeneic bone grafting materials are frequently used for bone augmentation. The effect of these materials on precursor cells for bone augmentation is yet to be determined. The aim of this study was to ascertain, in vitro, how augmentation materials influence the growth rates and viability of human unrestricted somatic stem cells. The biocompatibility of two xenogeneic and one alloplastic bone graft was tested using human unrestricted somatic stem cells (USSCs). Proliferation, growth, survival and attachment of unrestricted somatic stem cells were monitored after 24 h, 48 h and 7 days. Furthermore, cell shape and morphology were evaluated by SEM. Scaffolds were assessed for their physical properties by Micro-CT imaging. USSCs showed distinct proliferation on the different carriers. Greatest proliferation was observed on the xenogeneic carriers along with improved viability of the cells. Pore sizes of the scaffolds varied significantly, with the xenogeneic materials providing greater pore sizes than the synthetic inorganic material. Unrestricted somatic stem cells in combination with a bovine collagenous bone block seem to be very compatible. A scaffold’s surface morphology, pore size and bioactive characteristics influence the proliferation, attachment and viability of USSCs.

## 1. Introduction

Treatment of large bone defects remains a challenge in the field of invasive medicine. The choice of grafting material is highly patient-specific [1]. The current gold standard for the reconstruction and augmentation of critical-size defects is the use of autologous bone grafts [2]. The disadvantages of autologous bone grafts are their limited supply and frequent donor site morbidity [3]. Alternative treatments are applications of allogeneic, xenogeneic and alloplastic bone grafts. Their use might reduce treatment duration, reduce treatment costs and decrease patients’ morbidity. Xenogeneic bone substitutes are most common. They offer mainly osteoconductive properties [4]. Examples of bovine xenogeneic bone grafts are insoluble collagenous bone matrix (ICBM) and BioOss^®^ Collagen. Both have proven to be excellent materials for bone augmentation in previous studies [5,6]. ICBM mainly consists of collagen type I [7], whereas BioOss^®^ Collagen consists of a mixture of cancellous granules and collagen [8]. ICBM is predominately used in research settings [6,7], BioOss^®^ is often used in clinical routines [9,10]. Augmentative results of xenogeneic materials vary in literature [11,12]. Altered sensations over long periods of time after xenogeneic bone implantation have been described. A reason might be immune responses toward the materials [1,13].

Alloplastic bone substitute materials serve as a synthetic alternative to xenogeneic materials with osteoconductive properties. Calcium phosphate-based ceramics, such as hydroxyapatite, offer a high degree of biocompatibility [14]. NanoBone^®^ is a non-sintered nanocrystalline hydroxyapatite embedded in a highly porous silica gel matrix. Nano-hydroxyapatite is due to its biocompatibility and its resemblance to the inorganic bone structure used in implantology, surgery, periodontology, esthetics and prevention [15]. It is insoluble; therefore, the formation of new bone occurs only through the reabsorption of these hydroxyapatite particles. Small-sized HA crystals show great osteoconductive effects [1].

Materials for bone augmentation can be modified and enhanced regarding their compatibility and efficiency by the use of tissue engineering techniques. The application of growth factors, cytokines, scaffolds and cells leads to an improvement in bone and tissue regeneration as well as its growth [16]. ICBM, for example, has proven to enhance vertical peri-implant bone regeneration when combined with rhBMP2 + VEGF [6]. Tissue-engineered bone substitutes offer osteogenic, osteoconductive and osteoinductive properties. Stem cells, differentiated into osteogenic cells, provide the osteogenic potential. Unrestricted somatic stem cells (USSCs) derived from human umbilical cords can be differentiated into osteoblasts, chondroblasts, adipocytes, hematopoietic and neuronal cells. They show high proliferation and differentiation rates in vivo and in vitro [17]. USSCs can be converted into pluripotency by ectopic expression of OCT4, SOX2, KLF4 and C-MYC22 [18]. They show all characteristics of mesenchymal stem cells from bone marrow (BM-MSC) but offer a specific Hox-gene expression pattern resembling that of embryonic stem cells [19]. In addition, USSCs possess longer telomeres, exhibit a significantly lower senescence rate compared to BM-MSC, and do not form teratoma after transplantation [17]. USSCs have a regenerative phenotype influencing, at least partially, a multitude of relevant biological processes [20]. Their characteristics are still the focus of research [21]. Osteogenic pre-differentiated USSCs have been tested in combination with different biomaterials [22]. Osteogenesis begins with osteoinduction. During osteoinduction, basic, undifferentiated and pluripotent stem cells are recruited, stimulated and differentiated into preosteoblasts and osteoblasts [23,24]. Cell adhesion in general is altered by surface characteristics, such as roughness, phase content, porosity, crystallinity, solubility and surface energy [14,25]. If a certain constitution of carrier material affects growth rates and adhesion of unrestricted human stem cells in a positive or negative way is still uncertain.

The aim of this study was to investigate the biocompatibility of three clinically used bone substitute materials of different origins, i.e., ICBM (insoluble collagenous bone matrix), BioOss^®^ and NanoBone^®^, with USSCs in order to evaluate the carrier + USSC combination for their osteogenic potential and to assess the earliest stage of bone regeneration in particular. As an indicator of the influence of the different carrier materials both proliferation and growth behavior of unrestricted somatic stem cells were monitored. We hypothesized that the three-dimensional xenogeneic bone substitutes might offer better growth conditions for USSCs than alloplastic bone blocks.

## 2. Results

### 2.1. Proliferation

**Figure 1:**
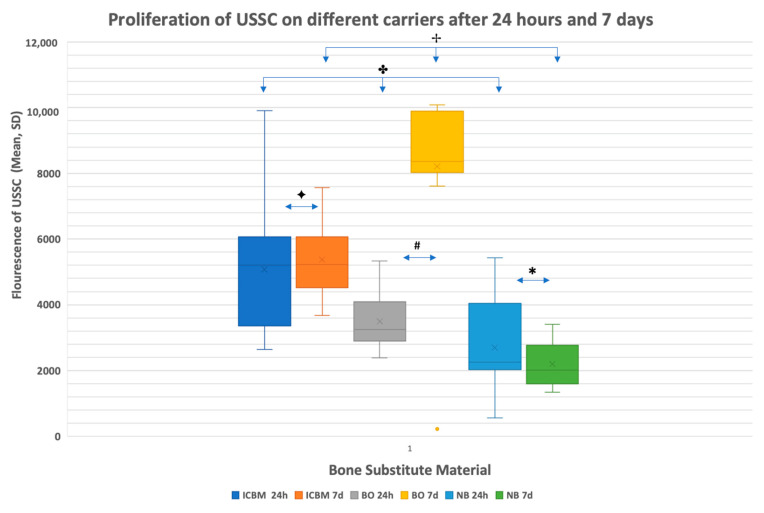
Mean fluorescence of unrestricted somatic stem cells (USSCs) after 24 h and after 7 days of cultivation including standard deviations. 24 replicates were measured for every material at each time point. Insoluble collagenous bone matrix (ICBM) 5078 (±1906) after 24 h and 5372 (±1153) after 7 days. BioOss (BO) 3490 (±797) after 24 h and 8212 (±2572) after 7 days. NanoBone (NB) 2689 (±1372) 24 h and 2204 (±617) after 7 days. ICBM and BO show greater cell count and proliferation rates than NO. BO shows highest proliferation rates after 7 days (ICBM, BO and NB after 24 h ✤ = *p* = 0.003, and ICBM, BO and NB after 7 d ✢ = *p* = 0.0015, ICBM 24 h/7 d ✦ = *p* = 0.048, BO 24 h/7 d # = *p* = <0.001, NB 24 h/7 d * = *p* = 0.017).

Results for proliferation varied significantly in the different groups after 24 hours and after 7 days (Figure 1). 

### 2.2. Cytotoxicity

Relative cytotoxicity in the ICBM group was 16.2 ± 1.2%. In the BO group, it was elevated with a mean of 23.91 ± 1.6% (1.5-fold higher than in the ICBM group). In the NB group, cytotoxicity was highest (29.7 ± 2.3%, 1.8-fold higher than in the ICBM group) (*p* = 0.0023).

### 2.3. SEM-Evaluation

**Figure 2:**
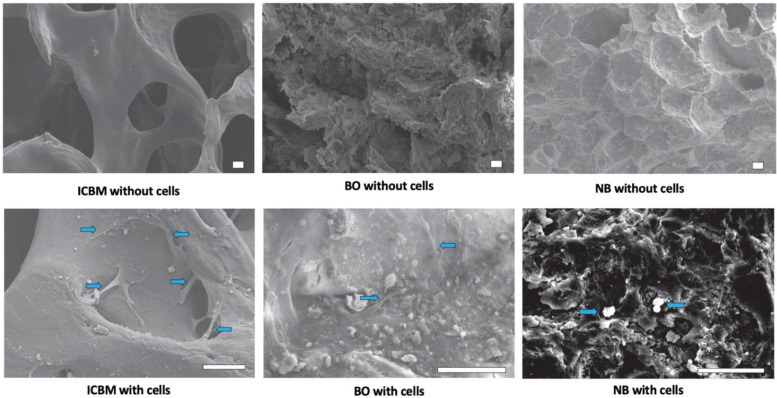
Scanning electron microscope images of the different carriers. In the upper panel, the carriers are displayed before cultivation with unrestricted somatic stem cells (USSCs). The lower panel shows the different carriers after 7 days of cultivation. USSCs in different shapes and sizes are visible (blue arrows). On the ICBM carrier cells show long ciliae as a sign of increased migratory activity. (white bar = 100 μm, upper panel farther away to gain a structural overview, lower panel zoomed in closer in order to detect and evaluate cells).

Empty ICBM carriers displayed wide perforations to enable cell in-growth. In comparison to the other two tested materials, ICBM carriers showed a smoother surface and a more spongious structure. Perforations in the BO group appeared smaller, and the surface of an NB scaffold appeared rough with small perforations (Figure 2). After 7 days of colonization with USSCs, a higher cell density was detected in the ICBM group than in the other groups. Furthermore, cells developed more cilia and more in-growth into the perforations on ICBM carriers than on BO or NB carriers. 

Out of the three carriers, NB showed the least cell-to-cell contacts and filopodia. In all carriers, USSCs seemed to be directly attached to the surface without major changes in their appearance.

### 2.4. Micro-CT Images

**Figure 3:**
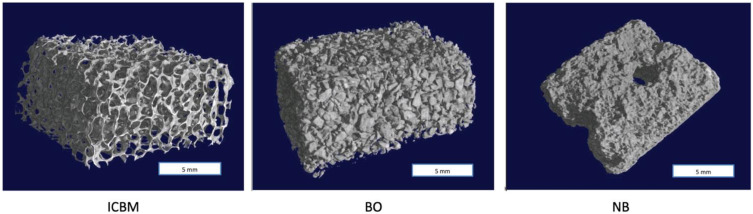
Micro-CT Images of the 1 × 1 × 0.5 cm^3^ blocks of three-dimensional biomaterials. Insoluble Collagenous Bone Matrix (ICBM) appears spongious with large pores. BioOss (BO) almost shows a particulate structure whereas NanoBone (NB) displays a smoother surface with only little pores.

When assessed by μCT major differences in the scaffolds emerged regarding their porosity and pore size. ICBM showed an increased porosity of 78.82% and large pores of around 487 μm in size. In BO a porosity of 62.61% was detected with a mean pore size of 243 μm. NB showed decreased porosity with 57.09% and the smallest mean pore size (219 μm) (*p* = 0.012) (Figure 3).

## 3. Discussion

The interaction between a biomaterial’s surface and its surrounding tissue determines a material’s impact on bone regeneration [26]. This study was designed to evaluate how the different three-dimensional carrier materials influence the proliferation and growth of unrestricted somatic stem cells. ICBM, compared to BO and NB, proved to be most suitable for the proliferation, growth and attachment of USSCs. 

The size, structure, surface and physicochemical properties of a scaffold biomaterial seem to be as important as its biochemical composition concerning its biocompatibility [27,28]. The topographic structure enables cell ingrowth, hydrophilic properties allow blood clot stabilization and the absorption of proteins facilitates the adhesion of osteoblasts [29]. Solid three-dimensional structures offer great volume stability over time. Bone blocks have proven superior to the use of paste or particulate material in terms of bone regeneration in vivo [30]. A three-dimensional collagen matrix in particular offers excellent conditions for osteoblast attachment, proliferation and differentiation [31]. As a result, in this study, three very different carriers in the constitution, solubility, particle size and pore size were evaluated. 

ICBM is a bovine-derived scaffold mainly consisting of collagen type 1. It has proven to provide excellent grounds for cell proliferation and growth [6,22]. BO is also bovine-derived, consisting of a mixture of spongious granules and collagen. A spongiosa-like structure of the material is achieved in the fabrication process leading to smaller micropores of 3–1.5 nm. In vivo, it offers great volume stability and remains insoluble [32]. Extraction sockets filled with BO show excellent maintenance of alveolar bone volume [33]. NB is a purely alloplastic material containing 61% nanocrystalline hydroxyapatite and 39% silica gel SiO_2_. It is fabricated in a special sol-gel procedure by which it gains higher porosity and remains softer than sintered hydroxyapatite materials. Granules are loosely packed to present a high porosity in order to enhance osteoinductive properties and degradability. Furthermore, the SiO_2_ promotes vascularization as well as collagen and bone growth [34].

In combination with the different carrier materials, USSCs showed distinct proliferation and attachment rates. The highest attachment rates were seen on the ICBM carrier after 24 h of incubation, followed by BO and NB showing slightly reduced attachment rates. The BO group which showed higher proliferation, showed intermediate cell death and intermediate cell density, compared to the other two materials. Intermediate pore sizes seem to offer great conditions for early proliferation. At a later stage, the supply of nutrients, as well as the removal of metabolites, becomes less efficient than in carriers with larger pores. As already stated, cell attachment strongly depends on the surface properties of the material. The spacious porous surface of the ICBM carrier seems to bring about higher attachment rates of the USSCs than the surfaces of BO and NB which appear less accessible for cell ingrowth. ICBM almost purely consists of collagen type I. In tissues, cells are anchored to collagenous structures through direct binding to the triple-helical domains or indirectly via matrix glycoproteins [35]. Therefore, the highly ordered fiber structure of collagen may on its own offer better conditions for USSC attachment than a mixture of materials, as is seen in the other materials tested. On BO highest proliferation rates were seen, whereas on NB cell counts were slightly reduced. Again, the ratio of collagen in each scaffold material might influence the proliferation rates, as collagen type I is known to enhance cell proliferation and osteogenesis of human mesenchymal stem cells [36]. A recent study compared BO and NB histologically, clinically and radiologically. In this study, BO induced a mild tissue reaction with only a few multinucleated giant cells and low vascularization, NB induced a multinucleated giant cell-triggered tissue reaction with an increase of vascularization as a sign of foreign body reaction [37]. Another decisive factor might be the characteristic porosity of each material [38]. Structural micro-CT analysis of the tested carriers focused especially on the materials’ microporosities. ICBM showed with 78.82% a high porosity with a very high proportion of large pore diameters with an average size of 487.8 μm. The ideal pore size of a carrier material for bone augmentation has been described as being between 300 and 700 μm [39,40]. The properties provided by ICBM offer excellent grounds for tissue integration and cell growth. BO and NO show smaller pore sizes of 243 μm and 219 μm. Consequently, their attachment properties are not offering the most favorable environment for USSCs. However, often smaller pore sizes provide a larger surface for the attachment of cells. Eweida et al. found that both a collagen matrix and a small particle size provided more favorable results in terms of vascularization and tissue formation than a combination of diluted fibrin and larger NB particles. In their study, the NB was tested with mesenchymal stem cells. Results showed decreasing cell viability within the first two weeks which finally recovered after three weeks [41]. NB seems to have an initial impairing effect on stem cells.

The morphological cell analysis in the SEM showed many cell-to-cell contacts and spindle-shaped dendritic protrusions, suggestive of increased migratory activity. On NB the USSCs kept a spherical shape and showed little cell-to-cell contacts. Similar results could be seen using osteogenically pre-differentiated USSCs and murine embryonal stem cells on different biomaterials. In this study, ICBM proved to be the most biocompatible biomaterial as well [22,42]. 

Cell toxicity of the different carriers was measured indirectly via fluorescence analysis. Results showed ICBM to be most biocompatible, again followed by BO. The synthetic NO presented as the least biocompatible and showed increased levels in the evaluation of toxicity. However, in comparison to other biomaterials, NO still showed a very moderate level of cell toxicity with a value of 29.7% dead cells in the testing [43]. A recent study compared BO and NB in a clinical trial. BO induced a mild tissue reaction with only a few multinucleated giant cells and low vascularization, NB induced a multinucleated giant cell-triggered tissue reaction with an increase in vascularization [37].

USSCs hold the potential for differentiation into osteoblasts, chondroblasts, adipocytes, hematopoietic and neuronal cells [17]. Therefore, they appear to be an especially promising cell type for tissue engineering. Furthermore, their regenerative phenotype might positively influence early bone regeneration [20]. Interestingly, a similar study investigating the biocompatibility of NB and BO using osteoblasts showed different results to our study, indicating the fact that osteoblasts have undergone significant changes in their ability to adjust to extrinsic materials. The synthetic NB promotes osteoblast proliferation slightly better than BO [43]. In the present study, however, BO shows higher proliferation rates and enhanced cell attachment in comparison to NO. The requirements for the biocompatibility of a material appear to be strongly cell-type-dependent and may also be connected to the level of differentiation of the cells themselves. Osteoblasts are a highly specialized cell type and seem to tolerate contact with inorganic surfaces better than undifferentiated cells. The current trend in research is to improve well-known dental materials by focusing on their physicochemical properties or adding growth factors and cells for augmentation [28,44,45]. A recent study successfully combined BO with concentrated growth factor gel for regeneration of cranial defect models in vivo. The gel contains several growth factors such as platelet-derived growth factor (PDGF), transforming growth factor-β (TGF-β), epidermal growth factor (EGF), bone morphogenetic protein-2 (BMP-2), vascular endothelial growth factor (VEGF) and insulin-like growth factor (IGF). [46]. A different study enhanced bone regeneration by adding only rh-BMP2 to BO [47]. The present study shows that stem cells such as USSCs in combination with a bovine collagenous bone block might enhance early human bone regeneration and augmentation. 

Among the limitations of the study is the short observation period. Osteogenic proliferation markers such as alkaline phosphatase are only detectable after 9–11 days [48]. Furthermore, a longer observational period might have shown a recovery of cells in the NO group [41]. However, cell attachment and proliferation are exceptionally important within the first week of healing. Although ICBM was the only insoluble carrier material in comparison to the other degradable materials, solubility is unlikely to influence results after only 7 days of evaluation. Another limitation is that only solid 3D carriers were investigated in this study. A solid scaffold makes it difficult to seed cells within the scaffold [14]. Furthermore, in vivo, high-volume stability and low degradability of a scaffold are essential for hard tissue augmentation. This applies in particular for lateral and vertical defect augmentation [49]. In vivo, therefore, a synthetic material might have performed better because of its lower degradability and greater volume stability. The choice of bone substitute material also depends on the desired purpose. Hydroxyapatite is a suitable bone graft candidate to reduce the high risk of donor morbidity and evoke less pain [50]. The results of this study cannot be transferred to in-vivo conditions. Under in vivo conditions, very limited amounts of stem cells are available and the use of human stem cells in an animal study may be biased due to immune reactions. A focus on the interaction between the human multipotent stem cells and the specific dental materials is only possible under in vitro conditions. However, the used dental biomaterials have already been tested in in vivo studies. Results show decent to good regenerative potential for all materials tested (ICBM [6], Bio Oss [5,33], Nanobone [37,51].

The perfect bone substitute material has yet to be found. In future research, other carriers could be tested for their augmentative potential with USSCs. The use of hydrogels, for example, might solve the cell seeding problem of solid carriers. Hydrogels can be 3D-bioprinted and might offer patient-specific augmentative options including growth factors, such as recombinant bone morphogenetic proteins (rhBMPs), in combination with USSCs. Promising results have recently been reported using other 3D scaffolds (i.e., bioprints) for bone augmentation in combination with precursor cells [52,53,54]. 

## 4. Materials and Methods

### 4.1. Set Up

In this study, the biocompatibility of three different bone grafts was tested using USSCs. Materials tested were:

1. ICBM: Insoluble collagenous bone matrix (ICBM, 90% bovine collagen, n = 24, n = 8 in each assay): bovine bone blocks produced after standardized protocol in our own lab [55,56];
2. BO: BioOss^®^ Collagen (n = 24, n = 8 in each assay): Deproteinized bovine bone mineral granules (90%), and bovine collagen (10%), Geistlich Biomaterials, Wolhusen, Switzerland
3. NB: NanoBone^®^ Block (n = 24, n = 8 in each assay): composite material made from nanocrystalline hydroxyapatite (61%) and silica gel SiO_2_ (39%), ARTROSS GmbH, Rostock, Germany.

After cell cultivation, the specimens were evaluated for cell proliferation (24 h and 7 days), and cytotoxicity (48 h). Furthermore, the specimens were morphologically and structurally examined before cultivation and evaluated for cell attachment after 7 days by scanning electron micrography (SEM). Furthermore, morphology and porosity of the uncultured scaffolds were evaluated by Micro-CT-Imaging. Empty controls were performed using the materials without cells.

This study was approved by the Ethics Committee of the Heinrich-Heine University Dusseldorf, Germany (No.: 2975). BioOss^®^ and NanoBone^®^ have already been tested in vivo for their approval as medical products.

### 4.2. Preparation of Carriers

The cuboid (1 × 1 × 0.5 cm^3^) ICBM carriers were produced as three-dimensional scaffolds on the basis of an already established patent [55] and procedure [7,56]. For ICBM production fresh, around 1 cm thick bovine femur bone slices were stored at −80° Celsius (°C). ICBM with medium cancellous bone density was used. The BO and NO were delivered ready to use and cut into the desired cuboid shapes. All bone grafts were sterilized using γRadiation (25 kGy, Gamma-Service Produktbestrahlung GmbH, Radberg, Germany). After 48 h of equilibration to medium conditions (medium was changed 3 times) USSCs were added to the specimens and cultured in an incubator. 

### 4.3. Cell Cultivation

Each tested material was inoculated with 500,000 cells contained in a 500µL medium. USSCs were provided by the José Carreras stem cell bank of the University of Duesseldorf. (Cell lines: USSC-18, (female, Passage 8), USSC-8 (female, Passage 9), USSC-8/17 (male, Passage 8)). The nutrient medium was Dulbecco’s Modified Eagle Medium (DMEM) low glucose (Lonza Cologne GmbH, Cologne, Germany) with 30% fetal calf serum (FCS, PAN-Biotech GmbH, Aidenbach, Germany), 1% penicillin/streptomycin (10,000 U/10,000µg/mL, Biochrom GmbH, Berlin, Germany) and 1% L-glutamine (200 mM, Biochrom GmbH, Berlin, Germany). Cells were precultivated in tissue culture dishes (100 mm × 20 mm style, Corning Inc., New York, NY, USA) and below 80% confluence harvested for inoculation of 24 well plates (Greiner, bio-one GmbH, Frickenhausen, Deutschland). Cells were cultivated in an incubator (37 °C, 21% O_2_ and 5% CO_2_ saturation). 

### 4.4. Proliferation

After 24 h and 7 days, specimens were carefully washed with attenuated PBS solution and frozen (–80 °C) until evaluation. Proliferation rates were determined with the help of CyQuant^®^ Cell Proliferation Assay Kit (Life Technologies GmbH, Darmstadt, Germany) following the manufacturer’s protocol. 

### 4.5. Cytotoxicity

Cytotoxicity was measured after 48 h from the release of lactate dehydrogenase to the cell culture supernatant using CytoTox-ONE™ Homogeneous Membrane Integrity Assays (Promega GmbH, Walldorf, Germany).

Toxicity was calculated according to manufacturer’s instructions. The average fluorescence values of the culture medium background were subtracted from all fluorescence values of experimental wells. Then the average fluorescence values from experimental, maximum LDH release and culture medium background were used to calculate the percent cytotoxicity:

$$relative\;cytotoxiciy=100\times \frac{\begin{array}{c} (Experimental-\\ Culture\;medium\;background) \end{array}}{\begin{array}{c} (Maximum\;LDH\;Release-\\ Culture\;medium\;background) \end{array}}$$

All USSCs on the carriers that were lysed provided the maximum value of cells in the analysis as this released the maximum achievable lactate dehydrogenase. 

### 4.6. SEM and Micro-CT Imaging

For scanning electron microscopy bone grafts were placed in 2.5% Glutardialdehyde for 3 h, dried by an ascending acetone series (50%-70%-80%-90%-100%) and fully dehydrated by critical point drying (model CPD 030 BAL-TEC GmbH, Schalksmuhle, Germany). Specimens were sputtered using Sputter Coater 108 auto (Ted Pella, Inc., Redding, CA, USA) and evaluated using a Scanning-Electron-Microscope S3000N (Hitachi High-Technologies Europe GmbH, Krefeld, Germany). Specimens were examined for surface structure, cell colonization, cell morphology and cell attachment before and 7 days after cultivation. In order to visualize the structure and porosity of the grafting materials in more detail, micro-CT Images of up to 0.7 μm slice thickness were taken (Micro-XCT, Xradia^®^ ZEISS, Oberkochen, Germany). Pore sizes were determined using a 3D object analyzer tool. Data were plotted as the mean pore size ± SD of the pore size distribution (measurements were performed at the Institute for Biomaterials, Technische Universität Dresden, Dresden, Germany).

### 4.7. Statistical Analysis

Sample sizes were based on a power analysis including an additional failure rate of 5% (A-priori power analysis, Effect-size 0.5, G*Power, Heinrich Heine University, Düsseldorf, Germany [57]). Data was blinded for statistical analysis. A Shapiro–Wilk test was used for the evaluation of normal distribution. Means and standard deviations were calculated. In order to detect statistically significant differences, a one-way ANOVA and the Bonferroni correction were performed as a post-hoc test. An analysis of variance (ANOVA) was executed to detect dependencies. *p* < 0.05 was set for a significant divergence. Calculations were performed by the use of SPSS 22 for Windows (SPSS Inc., Chicago, IL, USA).

## 5. Conclusions

In conclusion, this study shows that out of the tested 3D-carriers, scaffolds with a high collagen content turn out to be most compatible for tissue engineering using USSCs. The combination aims to enhance the very early stages of bone regeneration. Surface morphology, pore size and bioactive characteristics strongly influence the proliferation, attachment and viability of human USSCs. In this study ICBM proved best, followed by BO regarding the outcome of cell growth. Synthetic NO shows limited success for use in early USSC proliferation. Overall, ICBM and BO appear promising but further research has to follow in order to find the ideal combination for bone augmentation with USSCs.

## Data Availability

The datasets used and/or analysed during the study are available from the corresponding author on reasonable request.
